# Discovery and Preclinical Characterization of NE-2-6 as a Potent Thyroid Peroxidase Inhibitor with Antithyroid Activity

**DOI:** 10.3390/antiox15080967

**Published:** 2026-08-04

**Authors:** Min-Gyu Lee, Suzie Kang, Hyun-Jun Kang, Cheol-Won Yun

**Affiliations:** 1 School of Life Sciences and Biotechnology, Korea University, Seoul 02841, Republic of Korea; 2 EsgelBio Co., Seoul 02841, Republic of Korea

**Keywords:** thyroid peroxidase, antithyroid activity, Graves’ disease, propylthiouracil (PTU), thyroid-stimulating hormone receptor (TSHR)

## Abstract

Graves’ disease is an autoimmune hyperthyroid disorder in which thyroid peroxidase (TPO) plays a central role in excessive thyroid hormone production. However, current TPO-targeting drugs, such as propylthiouracil, have limited selectivity and can cause serious adverse effects. In this study, we established an integrated discovery pipeline to identify and optimize novel small-molecule TPO inhibitors. The pipeline began with high-throughput screening of approximately 7000 compounds from the KRICT chemical library for peroxidase inhibition, followed by cytotoxicity filtering and iterative medicinal chemistry to generate NE-2 derivatives. Lead compounds (NE-2-6, NE-2-7, and NE-2-8) were evaluated using enzymatic assays, selectivity profiling against myeloperoxidase (MPO) and lactoperoxidase (LPO), molecular docking, plasma pharmacokinetic profiling, and in vivo antithyroid pharmacodynamic testing in an Ad-TSHR289-induced thyroid hyperfunction model. NE-2-6 exhibited potent TPO inhibitory activity and relative selectivity within the tested peroxidase panel. UV–visible spectral scanning and H_2_O_2_-dependent inhibition assays suggested that NE-2-6 perturbs the heme-associated catalytic environment of TPO; however, these data do not establish a definitive binding mode or kinetic inhibition mechanism. In the Ad-TSHR289-induced thyroid hyperfunction model, NE-2-6 reduced serum T4 levels, supporting antithyroid pharmacodynamic activity. Because autoimmune endpoints and dose-matched PK–PD relationships were not fully assessed, the findings should be interpreted as preliminary preclinical evidence supporting further evaluation of NE-2-6 as a TPO-targeting antithyroid candidate.

## 1. Introduction

Graves’ disease is the most prevalent cause of autoimmune hyperthyroidism worldwide, characterized by the stimulatory action of autoantibodies on the thyroid-stimulating hormone receptor (TSHR), resulting in excessive synthesis and secretion of thyroid hormones (T3 and T4) and hypertrophy of the thyroid gland [1,2]. This condition leads to clinically significant manifestations, including weight loss, tachycardia, ophthalmopathy, and an increased risk of arrhythmia and osteoporosis [3]. The underlying pathogenesis involves dysregulation of multiple thyroid-specific factors, including TSHR, thyroglobulin (TG), and especially thyroid peroxidase (TPO), which catalyzes the iodination and coupling steps essential for hormone biosynthesis [4].

Standard therapeutic approaches—antithyroid drugs such as propylthiouracil (PTU) and methimazole—directly target the TPO enzyme to inhibit hormone production [5]. Although effective, these agents suffer from considerable drawbacks, including hepatotoxicity, agranulocytosis, limited selectivity, and frequent relapse following withdrawal [6,7]. Thus, there is a clear need for antithyroid therapies that combine potent, target-selective inhibition with improved safety profiles [8].

Recent progress in chemical library screening and medicinal chemistry has facilitated the exploration of novel small-molecule scaffolds with improved pharmacological characteristics [9]. In this work, we pursued an integrated pipeline to identify, optimize, and evaluate new TPO inhibitors through sequential in vitro, cellular, and in vivo studies. Using a primary screen of approximately 7000 compounds from the Korea Research Institute of Chemical Technology (KRICT) chemical bank, we used an MPO inhibition assay as an initial heme-peroxidase screening platform, followed by cytotoxicity filtering. Iterative medicinal chemistry produced NE-2 derivatives with structural modifications intended to improve potency and relative peroxidase selectivity.

We then performed subsequent profiling to assess TPO inhibition and relative selectivity over MPO and LPO. Cellular experiments involved SNU-790 human thyroid cells to assess compound-associated changes in TPO, TG, and TSHR gene expression. Finally, selected compounds were evaluated in an Ad-TSHR289-induced thyroid hyperfunction model to assess antithyroid hormone-lowering activity. Because autoimmune parameters such as anti-TSHR antibody titers, thyroid-infiltrating immune cells, and inflammatory cytokine profiles were not comprehensively assessed, the in vivo component of the present study was interpreted primarily as an evaluation of thyroid hyperfunction and antithyroid pharmacodynamic activity. Our data identify NE-2-6 as a potent TPO inhibitor candidate with selectivity within the tested peroxidase panel and preliminary short-term tolerability observations [10].

## 2. Materials and Methods

### 2.1. Enzyme Assays

Myeloperoxidase (MPO) inhibitory activity was assessed using a TMB/H_2_O_2_–based colorimetric assay [11]. Human recombinant MPO (10 µL) was incubated with test compounds followed by the addition of 80 µL of hydrogen peroxide (final concentration 0.75 mM) as the reaction initiator. The chromogenic substrate solution consisted of 110 µL of 2.9 mM TMB prepared in 14.5% DMSO and 150 mM sodium phosphate buffer (pH 5.4). The reaction mixture was incubated for 5 min at either 37 °C or room temperature to allow enzymatic turnover. The reaction was terminated by adding 50 µL of 2 M H_2_SO_4_, generating the characteristic yellow oxidized TMB product. Absorbance was measured at 450 nm using a microplate reader, and MPO activity was quantified relative to vehicle-treated controls. TPO activity was quantified using a luminol-based chemiluminescence assay. Cellular lysates (0.1–0.2 mg/mL protein) were prepared in reaction buffer containing 1 M glycine-NaOH (pH 9.0) and 1 mM EDTA. Test compounds were added at 1/100 of the reaction volume and gently mixed. The samples were incubated at 37 °C for 30 min with continuous gentle agitation to allow compound–enzyme interaction. Following preincubation, TPO activity was initiated by adding 20 µL of luminol reaction mixture comprising 1 M glycine-NaOH (pH 9.0), 1 mM EDTA, and 400 µM luminol. The oxidative reaction was triggered by adding 5 µL of 80 mM H_2_O_2_. To further examine the inhibition behavior of NE-2-6, TPO activity was measured under the standard 30 min reaction condition and a prolonged 2 h reaction condition. IC_50_ values were calculated by nonlinear regression from concentration-response curves. To evaluate whether NE-2-6 inhibition was overcome by excess H_2_O_2_, TPO inhibition was also measured at two H_2_O_2_ concentrations of 40 and 120 µM, under otherwise comparable assay conditions. These experiments were designed to test whether NE-2-6 behaves as a simple H_2_O_2_-competitive inhibitor and to assess whether inhibition changes during sustained TPO/H_2_O_2_ turnover. Chemiluminescence generated through the TPO-catalyzed oxidation of luminol was measured as relative luminescence units (RLUs) integrated over 10 s using a Luminoskan Ascent, Thermo Labsystems, Vantaa, Finland. Enzymatic activity was expressed relative to vehicle-treated controls.

### 2.2. Synthesis and Characterization of NE-2 Derivatives

NE-2 derivatives were synthesized as follows. Briefly, a benzodioxole-based cyano intermediate was prepared from the corresponding aminobenzodioxole starting material. The aminobenzodioxole derivative (8.7 g, 62.7 mmol) was treated with ethyl formate, followed by activation with POCl_3_ in the presence of triethylamine in THF, to afford the cyano-substituted benzodioxole intermediate (17 g, 114 mmol) in approximately 85% yield. For the synthesis of NE-2-6, the cyano intermediate was condensed with pyridinecarboxaldehyde (5.5 g, 50.9 mmol) and an aminopyridine derivative (5.3 g, 48.4 mmol) in the presence of scandium triflate [Sc(OTf)_3_] in CH_2_Cl_2_/MeOH. The reaction afforded the final tricyclic benzimidazole-pyridine derivative NE-2-6 in approximately 60% yield. The isolated product had a molecular weight of 344.37 and was obtained as 10 g (29.1 mmol). For the synthesis of NE-2-7, the same cyano intermediate was reacted with a methoxy-substituted benzaldehyde (15 g, 109 mmol) and an aminopyridine derivative (11 g, 103 mmol) under Sc(OTf)_3_-catalyzed conditions in CH_2_Cl_2_/MeOH, yielding NE-2-7 in approximately 60% yield. NE-2-8 was prepared by demethylation of NE-2-7 with boron tribromide (BBr_3_) in CH_2_Cl_2_, converting the methoxy group to the corresponding hydroxyl group. The reaction afforded NE-2-8 in approximately 80% yield. The isolated NE-2-8 product had a molecular weight of 359.39 and was obtained as 10 g (27.9 mmol). The identities of the synthesized NE-2 derivatives were confirmed by ^1^H NMR, ^13^C NMR, and mass spectrometry, and compound purity was assessed by analytical HPLC or LC-MS before biological evaluation.

### 2.3. Molecular Docking

Molecular docking analysis was performed using the CB-Dock2 program (https://cadd.labshare.cn/cb-dock2/index.php accessed date: 5 February 2026) to predict potential binding poses and interactions of NE-2-6 and NE-2-7 with human thyroid peroxidase (TPO; UniProt accession P07202) [12]. The three-dimensional TPO structure associated with UniProt accession P07202 was prepared by removing extraneous molecules and adding hydrogen atoms. The ligand structures (NE-2-6 and NE-2-7) were prepared by optimization and conversion to the appropriate format. CB-Dock2 identified candidate binding cavities and generated multiple docking poses.

### 2.4. UV–Visible Spectral Scanning of TPO

To evaluate whether NE-2-6 perturbs the catalytic environment of TPO, UV–visible spectral scanning was performed over 320–430 nm, a wavelength range that includes the heme-associated Soret region of peroxidase enzymes. Recombinant human TPO was purchased from KACTUS Bio (Waltham, MA, USA). For heme reconstitution, hemin solution was added dropwise to apo-TPO at a 3-fold molar excess (3:1 molar ratio of hemin to apo-TPO) with gentle agitation to minimize nonspecific protein precipitation. The mixture was incubated overnight at 4 °C in the dark to allow heme incorporation. Following incubation, unreacted and excess free hemin was removed using a Sephadex G-25 desalting column (PD-10, GE Healthcare, Chicago, IL, USA) equilibrated with the reconstitution buffer. The eluted holo-TPO fraction was collected, and heme incorporation was assessed spectrophotometrically by monitoring the characteristic Soret-band absorbance at 411 nm. Holo-TPO was prepared in phosphate-buffered saline (PBS, pH 7.4) and incubated with PTU or NE-2-6 at the indicated concentrations. Control samples contained human TPO in PBS without compound. UV–visible absorbance spectra were recorded from 280 to 500 nm. To evaluate compound-associated changes in the heme-associated Soret region, spectra were further analyzed from 330 to 430 nm. Compound-induced absorbance loss was calculated by subtracting the absorbance spectrum of each compound-treated sample from that of the corresponding untreated TPO control:

−ΔA = Acontrol−Acompound-treated(1)

The maximum −ΔA value within the Soret region was defined as the apex −ΔA, and the corresponding wavelength was recorded as the apex wavelength. PTU and NE-2-6 were compared at matched compound concentrations.

### 2.5. RNA Extraction and Quantitative PCR

Total RNA was extracted from SNU-790 human thyroid cells and spleen tissues using the RNeasy Mini Kit (Qiagen, Hilden, Germany) according to the manufacturer’s instructions. Briefly, samples were homogenized in RLT buffer, and RNA was isolated by column purification. RNA quantity and integrity were assessed using a NanoDrop spectrophotometer (Thermo Fisher Scientific, Waltham, MA, USA) and agarose gel electrophoresis, respectively. cDNA was synthesized using the iScript cDNA Synthesis Kit (Bio-Rad, Hercules, CA, USA), and quantitative real-time PCR (RT-qPCR) was performed using SYBR Green Master Mix (Thermo Fisher Scientific, Waltham, MA, USA) on a real-time PCR system. Gene-specific primers were used for TPO, TSHR, and TG, with GAPDH as the internal control. Relative gene expression levels were calculated using the 2^−ΔΔCt^ method. Primer sequences are provided in Table 1.

### 2.6. Construction of the Ad-TSHR289-Induced Mouse Model and Measurement of Serum Thyroxine Levels

To construct the TSHR289-expressing vector, the backbone plasmid pAV[Exp]-CMV/EGFP, based on the Ad5 gene expression system, was used as an empty control vector. A DNA fragment encoding amino acids 1–289 of the human TSH receptor, which includes nearly the entire A-subunit, was inserted into this vector to generate pAV[Exp]-EGFP-CMV/{TSHR289}, as reported earlier [13]. Both vector construction and adenoviral packaging were performed by VectorBuilder (Chicago, IL, USA) using their custom adenovirus production service. Six-week-old female BALB/c mice (DooYeol Biotech, Seoul, Republic of Korea) were injected i.m. with Ad/TSHR 10^11^ particles or Ad/Empty vector 10^11^ particles in 50 µL PBS. Mice were injected three times at 3-week intervals. To evaluate the onset and severity of hyperthyroidism, serum thyroxine (T4) levels were measured in each experimental group following the second immunization.

This assessment provided a quantitative indicator of thyroid hyperfunction in the Ad-TSHR289-immunized mouse model. Total serum thyroxine (T4) levels were quantified using an enzyme immunoassay kit (Arbor Assays, Ann Arbor, MI, USA) according to the manufacturer’s instructions. Briefly, 10 µL of standards and serum samples diluted 1:800 in assay buffer were added to each well, whereas 35 µL of assay buffer was added to the nonspecific binding (NSB) well. Subsequently, 25 µL of conjugate solution was added to all wells, followed by 25 µL of antibody solution to all wells except the NSB well. Plates were incubated for 1 h at room temperature with gentle shaking, washed four times with 250 µL of wash buffer, and blotted dry. TMB substrate (100 µL) was then added to each well and incubated for 10 min, after which 50 µL of stop solution was added. Absorbance was measured at 450 nm using a SpectraMax (San Jose, CA, USA) plate reader, and T4 concentrations were calculated using a four-parameter logistic (4PL) curve fit. The intra-assay and inter-assay coefficients of variation (CVs) were 3.0% and 6.3%, respectively, as provided by the manufacturer.

### 2.7. Pharmacokinetic Analysis

Male ICR mice (5–6 weeks old; approximately 20 g) were used for pharmacokinetic analysis (*n* = 3 per time point). NE-2-6 was formulated in an appropriate vehicle and administered orally as a single 10 mg/kg dose. Blood samples were collected at predetermined time points after dosing. Plasma was separated by centrifugation and stored at −80 °C until analysis. Plasma concentrations of NE-2-6 were quantified using a validated UHPLC-MS/MS method. Pharmacokinetic parameters were calculated at the Nonclinical Research Center, QuBEST BIO (Seoul, Republic of Korea). Noncompartmental analysis of plasma NE-2-6 concentrations was performed using Phoenix^®^ WinNonlin^®^ software (version 8.3; Certara, Radnor, PA, USA).

### 2.8. Drug Treatment of SNU-790 Cells and Ad-TSHR289-Induced Thyroid Hyperfunction Model

Cell viability was assessed using SNU-790 cells. Cells were seeded in 96-well plates at 1 × 10^4^ cells per well and allowed to attach overnight. PTU and NE-2 derivatives were added at the indicated concentrations and incubated for 24 h. MTT solution (5 mg/mL) was then added to allow formazan formation. After removal of the medium, the formazan crystals were dissolved in DMSO, and absorbance was measured at 570 nm with a 630 nm reference wavelength. Viability was calculated relative to untreated controls.

To evaluate antithyroid pharmacodynamic activity in vivo, Ad-TSHR289-immunized mice were randomly assigned to treatment groups receiving vehicle, PTU, NE-2-6, or NE-2-7 at the indicated doses. Compounds were administered orally once daily for three weeks. Following the final administration, blood samples were collected and serum total T4 concentrations were measured using a commercial assay. Group differences in serum T4 levels were analyzed by one-way ANOVA followed by Dunnett’s multiple-comparison test against the Ad-TSHR289 vehicle group unless otherwise specified. This model was used to assess thyroid hyperfunction and hormone-lowering activity; autoimmune disease-modifying endpoints were not comprehensively evaluated in the present study.

### 2.9. Statistical Analysis

Data are presented as mean ± SD unless otherwise indicated. For in vivo serum T4 measurements, multiple treatment groups were compared using one-way ANOVA followed by Dunnett’s multiple-comparison test against the Ad-TSHR289 vehicle group. For in vitro RT-qPCR and cell-viability experiments, one-way ANOVA followed by Dunnett’s multiple-comparison test was used, with each treatment group compared with the corresponding untreated or vehicle control. Because the short-term body-weight study did not include a vehicle-only control group, body-weight data for the 300 and 600 mg/kg groups were interpreted descriptively as a preliminary dose-comparison observation. A *p* value < 0.05 was considered statistically significant. The number of biological replicates or animals per group is stated in each figure legend.

## 3. Results

### 3.1. Primary Screening of Myeloperoxidase Inhibitors and Selection of NE-2

**Figure 1:**
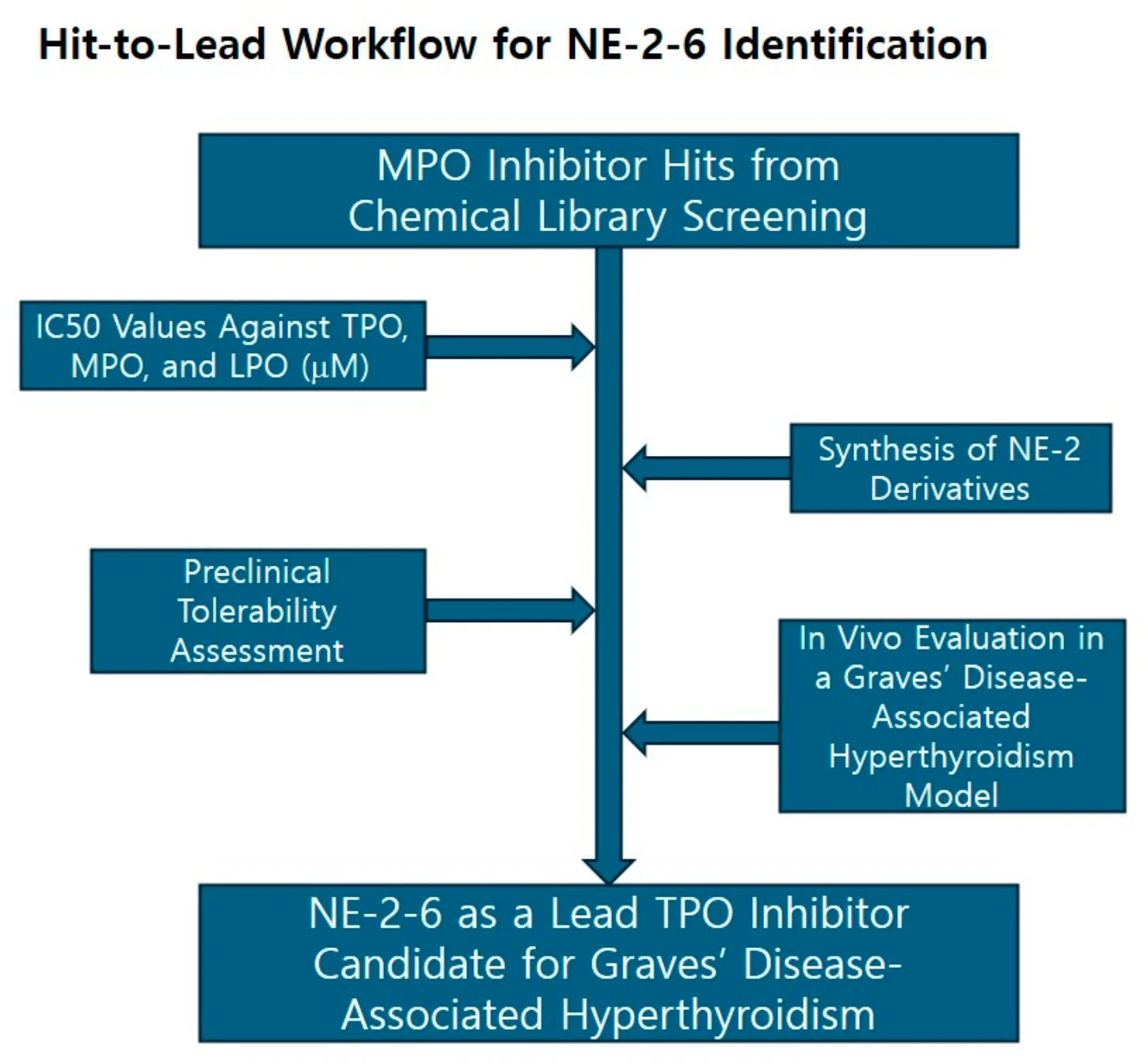
Hit-to-lead decision tree for the second-round optimization of NE-2 derivatives. The schematic illustrates the workflow used to advance the NE-2 derivative series toward lead identification. Initial lead candidates underwent systematic synthetic modification to generate NE-2 derivatives. In parallel, in vitro potency was evaluated by determining IC_50_ values against MPO, LPO, and TPO. Subsequent in vivo evaluation focused on antithyroid pharmacodynamic activity in an Ad-TSHR289-induced thyroid hyperfunction model. Integrated evaluation of potency, peroxidase selectivity, preliminary cytotoxicity, and short-term tolerability observations led to the selection of NE-2-6 for further preclinical evaluation.

A total of approximately 7000 chemical compounds from the KRICT Chemical Bank (www.chembank.org accessed date: 22 September 2022) were screened for MPO inhibitory activity, resulting in the identification of 16 active compounds as shown in Figure 1. In the second-round screening, these compounds (NE-1 to NE-16) were compared with the reference inhibitor AZD3241, yielding IC_50_ values ranging from 0.32 to 2.0 μM [14]. NE-8 (0.32 μM), NE-13 (1.02 μM), and NE-4, NE-6, NE-7, and NE-14 (approximately 1.5 μM) showed the lowest IC_50_ values. Several active compounds, including NE-2, NE-4, NE-6, NE-7, NE-8, NE-13, and NE-14, showed minimal cytotoxicity (0–5% relative cell death at 50 μM in HEK293T cells) and strong MPO inhibition (70–98%) in HL-60 cell lysates, whereas no inhibition was detected in the culture medium, suggesting that the inhibitory activity was associated with the cellular lysate (Table 2). Based on these findings, NE-2 was selected as the lead scaffold for further optimization.

### 3.2. Synthesis of NE-2 Derivatives and Hit-to-Lead Optimization

**Figure 2:**
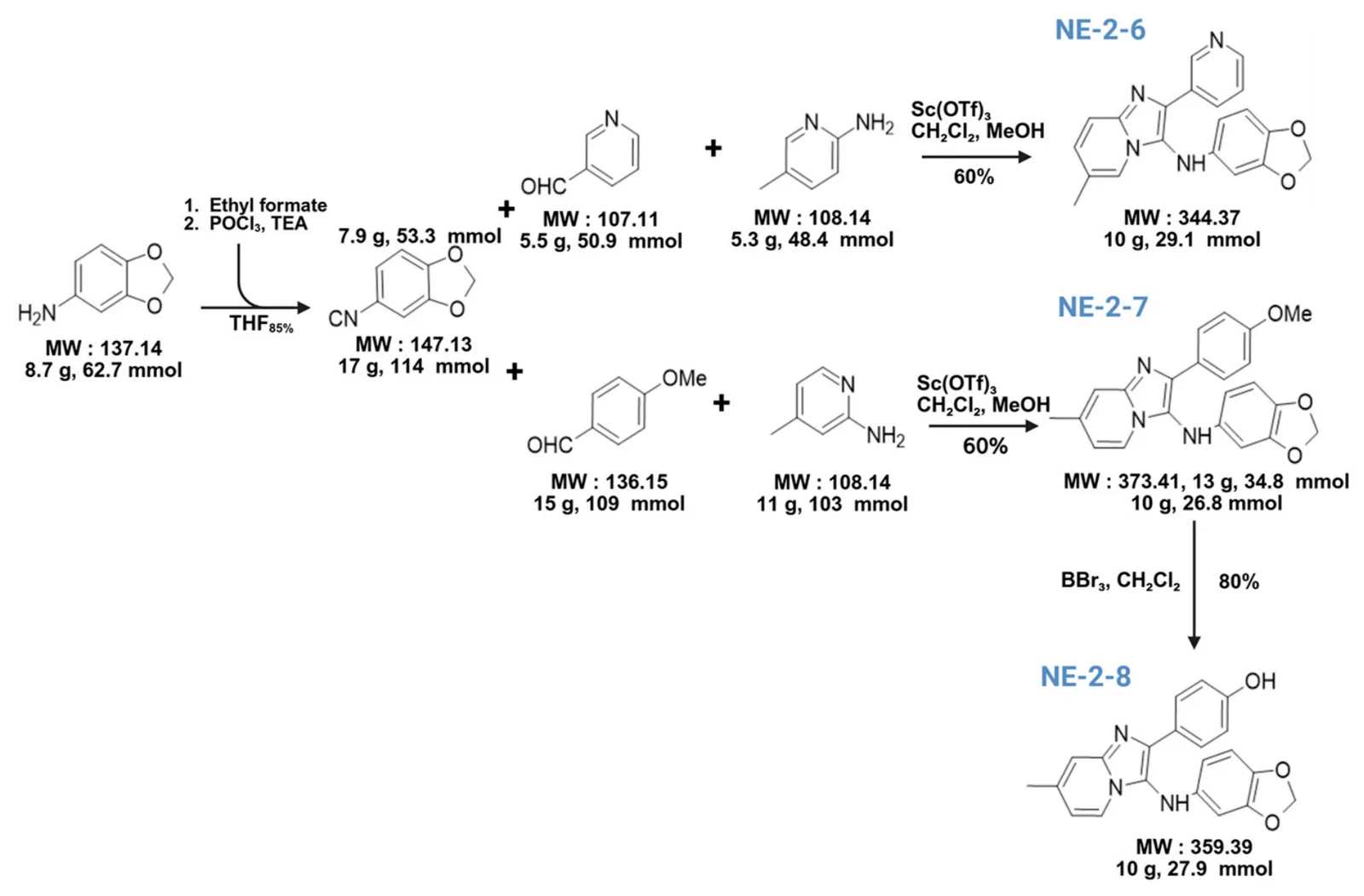
Synthetic scheme for the preparation of NE-2 derivatives and final lead candidates (NE-2-6, NE-2-7, and NE-2-8). The synthetic pathway toward the NE-2 derivatives begins with the formylation of the starting aminobenzodioxole (MW 137.14) using ethyl formate followed by POCl_3_/TEA activation to afford the cyano intermediate (MW 147.13, 85% yield). Condensation of this cyano intermediate with substituted benzaldehydes and 2-aminopyridine derivatives in the presence of Sc(OTf)_3_ (CH_2_Cl_2_/MeOH) produced key heterocyclic scaffolds. Reaction of pyridyl aldehyde (MW 107.11) and aminopyridine (MW 108.14) generated the final compound NE-2-6 (MW 344.37, 60% yield). Similarly, the methoxy-substituted aldehyde (MW 136.15) with aminopyridine afforded NE-2-7 (MW 373.41, 60% yield), which was subsequently demethylated using BBr_3_ in CH_2_Cl_2_ to provide NE-2-8 (MW 359.39, 80% yield). These three derivatives were selected as final lead candidates based on chemical tractability and biological evaluation.

Based on the initial screening results, a series of NE-2 derivatives was synthesized for lead optimization and subsequent pharmacological evaluation (Figure 2). The synthetic strategy aimed to improve TPO inhibitory potency, relative peroxidase selectivity, physicochemical properties, and cellular compatibility. The route began with preparation of a benzodioxole-based cyano intermediate from the corresponding aminobenzodioxole precursor using ethyl formate and phosphoryl chloride (POCl_3_) in the presence of triethylamine. This intermediate was subsequently condensed with selected aldehydes and aminopyridines under Sc(OTf)_3_-catalyzed conditions in CH_2_Cl_2_/MeOH to yield the tricyclic benzimidazole-pyridine derivatives NE-2-6 and NE-2-7 in approximately 60% yield. NE-2-7 was then demethylated with boron tribromide (BBr_3_) in CH_2_Cl_2_ to afford NE-2-8 in 80% yield. The resulting derivatives, NE-2-6, NE-2-7, and NE-2-8, were generated as lead candidates for subsequent biochemical, docking, cellular, pharmacokinetic, and in vivo evaluation.

An exploratory structure–activity relationship (SAR) analysis was performed using NE-2 and its derivatives to examine how structural modifications affected peroxidase inhibitory activity. The parent compound NE-2 contains a fused tricyclic benzimidazole-pyridine scaffold that provides a planar heteroaromatic framework for further modification. To explore the effects of polarity and substituent identity, three derivatives were evaluated (Table 3). NE-2-6, corresponding to the upper-right final product in Figure 2, incorporates a pyridyl substituent. NE-2-7 contains a methoxy-substituted phenyl group, whereas NE-2-8 was generated by demethylation of NE-2-7 to introduce the corresponding hydroxyl group. These modifications were intended to modulate polarity, lipophilicity, and potential interactions within the TPO catalytic environment. Because the number of derivatives was limited, these data were interpreted as an exploratory SAR comparison rather than a quantitative SAR model.

Enzyme inhibition profiling across the peroxidase family—lactoperoxidase (LPO), myeloperoxidase (MPO), and thyroid peroxidase (TPO)—showed enzyme-dependent inhibitory patterns among the tested compounds. The initial MPO assay served as a heme-peroxidase screening platform for chemical triage, whereas subsequent profiling directly assessed TPO inhibition and relative selectivity over MPO and LPO. NE-2-6 exhibited the strongest TPO inhibition among the NE-2 derivatives, with an IC_50_ of 0.013 µM under the assay conditions used. Selectivity ratios were calculated as the IC_50_ for MPO or LPO divided by the IC_50_ for TPO. NE-2-6 showed approximately 496-fold selectivity for TPO over MPO and 68-fold selectivity for TPO over LPO. These results indicate a pronounced preference for TPO inhibition within this limited enzyme panel. However, selectivity against MPO and LPO should not be interpreted as evidence of systemic safety or broad off-target selectivity. Collectively, these data identify NE-2-6 as a potent TPO inhibitor for further biochemical and pharmacological evaluation (Table 3).

### 3.3. Docking-Based Structural Hypothesis and Biochemical Evaluation of NE-2-6-Mediated TPO Inhibition

**Figure 3:**
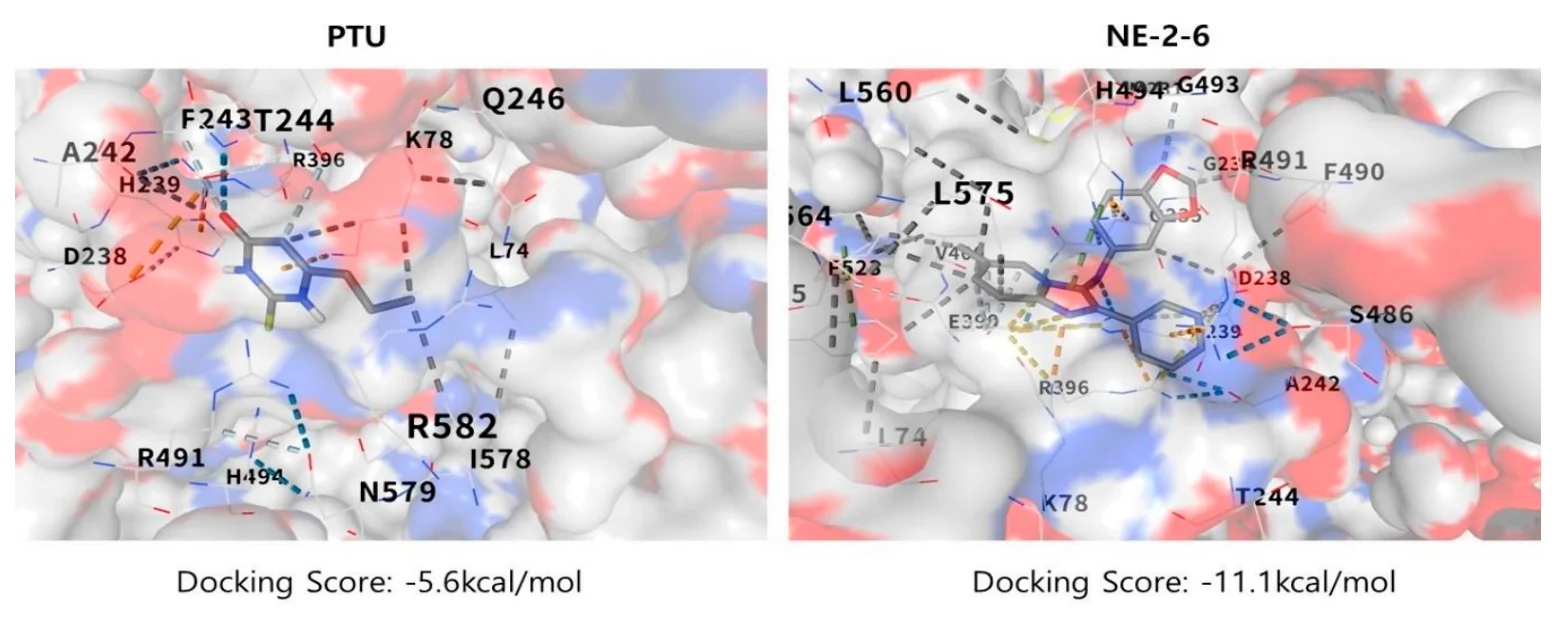
Comparative molecular docking analysis of PTU and NE-2-6 binding to thyroid peroxidase (TPO). The (**left** panel) shows the docking-predicted pose of PTU, and the (**right** panel) shows the docking-predicted pose of NE-2-6 in the TPO model. The protein surface is displayed with electrostatic coloring, and the ligands are shown as stick models. Residue labels indicate amino acids located near the predicted ligand pose; residue numbering follows the human TPO sequence used for the docking model. The panels are oriented to show the catalytic-channel region viewed from the pocket entrance toward the heme-associated catalytic area. Dashed lines indicate predicted polar contacts or hydrogen-bonding interactions. Docking scores and poses are presented only as computational structural hypotheses and should not be interpreted as quantitative binding-affinity measurements or definitive evidence of a specific allosteric or non-heme mechanism.

Molecular docking was performed to generate structural hypotheses for the interactions of PTU and NE-2-6 with TPO. As shown in Figure 3, the docking model placed PTU deep within the heme-containing catalytic cavity and predicted contacts with residues near the active-site environment. By contrast, the docking model placed NE-2-6 in a broader hydrophobic pocket near the entrance of the catalytic channel, with predicted interactions involving F490, S486, H491, E390, R396, and L575. These computationally predicted poses suggest different possible orientations of PTU and NE-2-6 within the TPO model. However, docking alone cannot establish a specific binding site, direct or indirect heme coordination, an allosteric mechanism, or the kinetic mode of inhibition. Accordingly, the docking results are presented only as structural hypotheses requiring further experimental validation.

**Figure 4:**
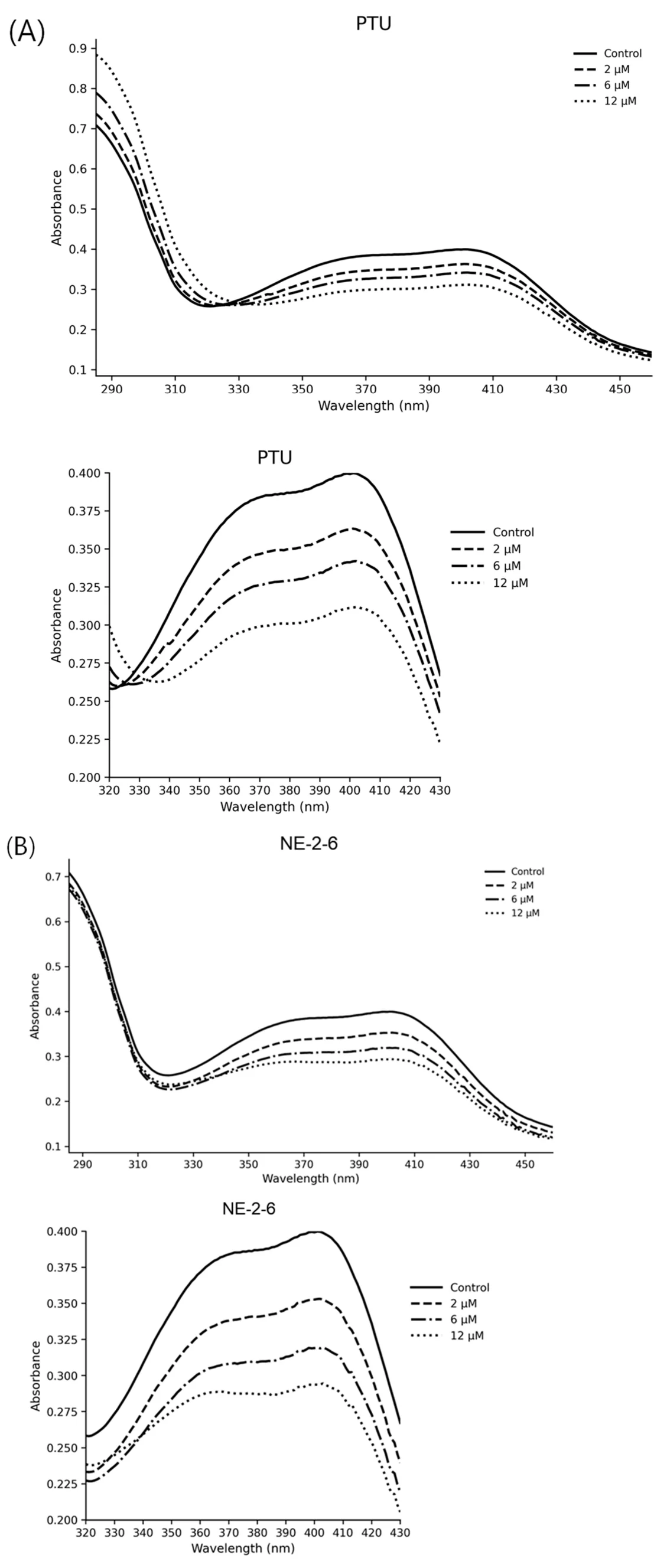

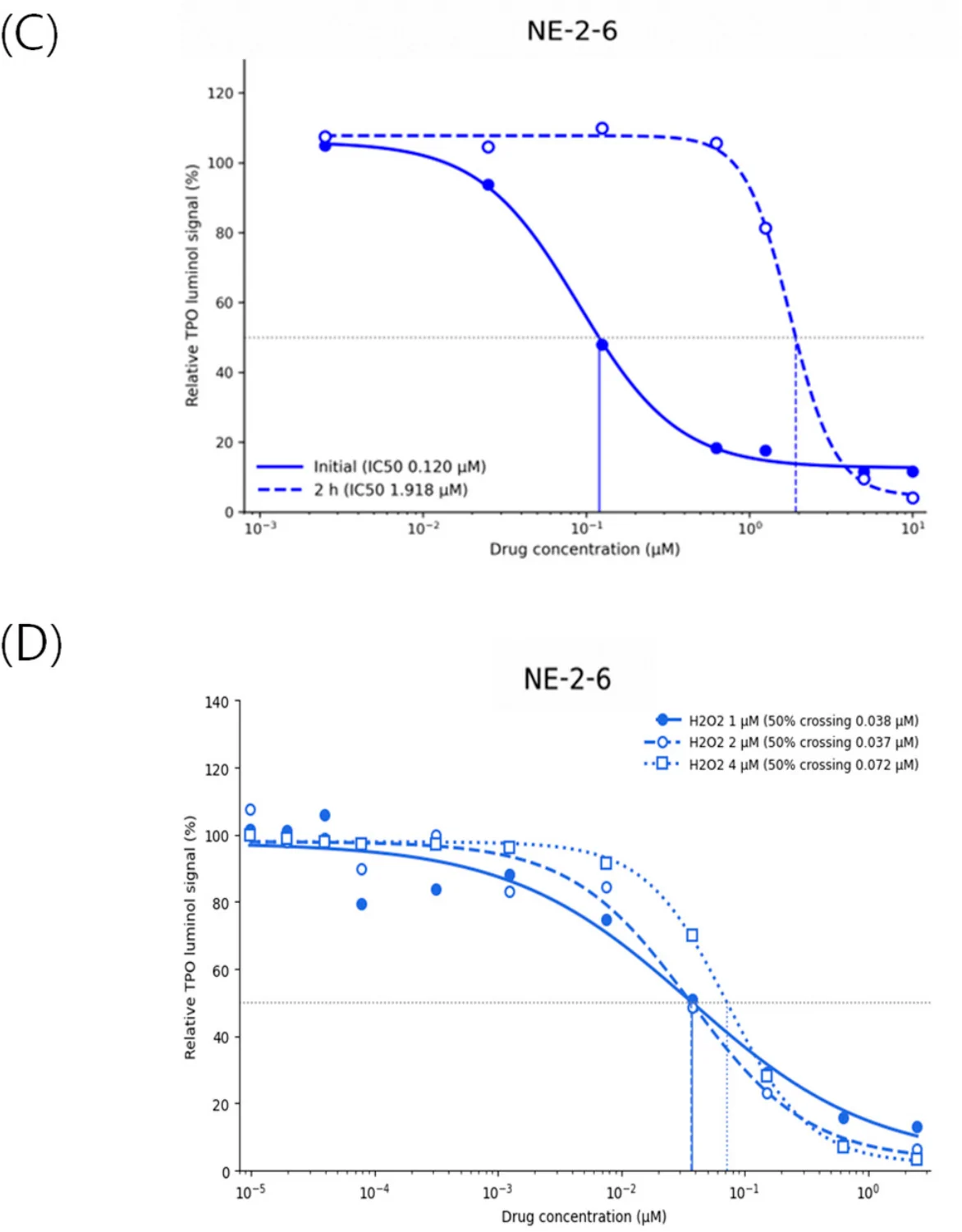
Biochemical characterization of NE-2-6-mediated TPO inhibition. (**A**) UV–visible spectral scanning of TPO treated with PTU at 2, 6, and 12 µM. PTU reduced the TPO-associated absorbance profile across 330–430 nm. (**B**) UV–visible spectral scanning of TPO treated with NE-2-6 at 2, 6, and 12 µM. NE-2-6 induced a concentration-dependent reduction in the TPO-associated absorbance profile, including the heme-associated Soret region. (**C**) Reaction-time-dependent TPO inhibition by NE-2-6. NE-2-6 showed potent initial inhibition with an IC_50_ of approximately 0.120 µM, whereas a 4-fold longer reaction shifted the IC_50_ to approximately 1.918 µM. (**D**) Effect of H_2_O_2_ concentration on NE-2-6-mediated TPO inhibition. Increasing H_2_O_2_ from 40 to 120 µM did not increase the IC_50_ of NE-2-6, suggesting that inhibition is not readily overcome by excess H_2_O_2_. These data indicate perturbation of the heme-associated catalytic environment, while the precise kinetic mode remains unresolved.

To obtain biochemical information beyond docking, UV–visible spectral scanning was performed over 320–430 nm. Both PTU and NE-2-6 altered the TPO-associated absorbance profile in the heme/Soret-region range, indicating perturbation of the heme-associated microenvironment or redox state of TPO (Figure 4A,B). However, these spectral changes do not distinguish direct heme coordination from indirect conformational or redox effects and should not be interpreted as evidence that NE-2-6 adopts either a PTU-like or a distinct binding mode. Thus, the docking and spectral results are presented as supportive biochemical observations rather than conclusive evidence of a novel binding mechanism.

We next examined whether NE-2-6-mediated TPO inhibition changed during prolonged catalytic turnover. Under the standard 30 min reaction condition, NE-2-6 inhibited TPO with an apparent IC_50_ of approximately 0.120 µM. When the reaction time was extended to 2 h, the apparent IC_50_ shifted to approximately 1.918 µM. This reduction in apparent potency during prolonged turnover argues against progressive irreversible TPO inactivation under the tested conditions (Figure 4C). We also examined whether increasing H_2_O_2_ concentration could overcome NE-2-6-mediated inhibition. Increasing H_2_O_2_ from 40 to 120 µM did not markedly shift the apparent IC_50_ of NE-2-6 under the assay conditions used (Figure 4D), suggesting that inhibition was not readily overcome by excess H_2_O_2_. However, these endpoint IC_50_ experiments do not define whether NE-2-6 is competitive, noncompetitive, uncompetitive, or mixed with respect to individual TPO substrates. Full steady-state kinetic analysis will be required to determine the precise inhibition mechanism.

### 3.4. Effects on Thyroid-Related Gene Expression In Vitro

**Figure 5:**
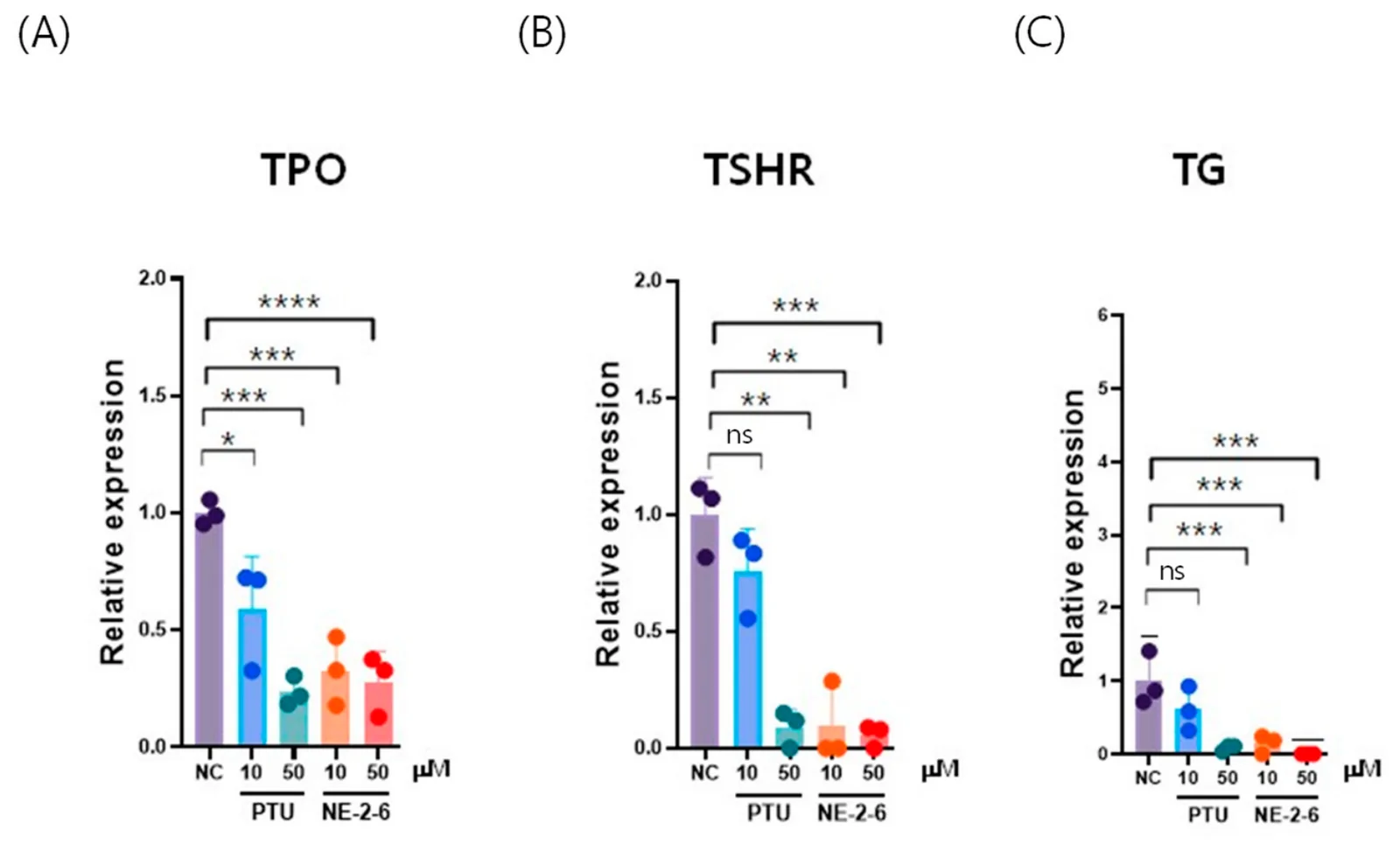
Effects of PTU and NE-2 derivatives on the expression of thyroid-related genes in vitro. SNU-790 cells were treated for 24 h with PTU (10 or 50 µM) or NE-2-6, NE-2-7, and NE-2-8 (10 or 50 µM), and the mRNA levels of (**A**) thyroid peroxidase (TPO), (**B**) thyroid-stimulating hormone receptor (TSHR), and (**C**) thyroglobulin (TG) were quantified by qRT-PCR. Relative expression was normalized to GAPDH and expressed as fold change relative to the untreated control (NC). Bars indicate mean ± SD of *n* = 3 independent experiments, and dots represent individual values. * *p* < 0.05, ** *p* < 0.01, *** *p* < 0.001 vs. NC (one-way ANOVA followed by Dunnett’s multiple-comparison test). * *p* < 0.05, ** *p* < 0.01, *** *p* < 0.001 **** *p* < 0.0001; ns, not significant (one-way ANOVA with appropriate post hoc test).

To evaluate compound-associated cellular responses, RT-qPCR analysis was performed in SNU-790 human thyroid cells treated with PTU or NE-2 derivatives (Figure 5). Each compound was tested under conditions that did not cause overt cytotoxicity in the MTT assay. NE-2-6 treatment was associated with reduced TPO mRNA expression relative to untreated control cells, and this reduction was more pronounced than that observed with PTU under the same experimental conditions. TSHR and TG mRNA expression levels were also evaluated and are presented in Figure 5.

These RT-qPCR findings are interpreted as compound-associated changes in thyroid-related gene expression and should not be taken as definitive evidence that NE-2-6 directly represses TPO transcription. TPO expression may be influenced by thyroid-cell differentiation status, redox balance, cellular stress, and feedback regulation. Additional studies assessing TPO protein abundance, cellular TPO enzymatic activity, promoter activity, thyroid differentiation markers, and stress-response pathways will be required to determine whether the observed mRNA changes reflect direct transcriptional regulation or secondary cellular adaptation.

### 3.5. Preliminary Cytotoxicity, Short-Term Tolerability, and Plasma Pharmacokinetics of NE-2 Derivatives

**Figure 6:**
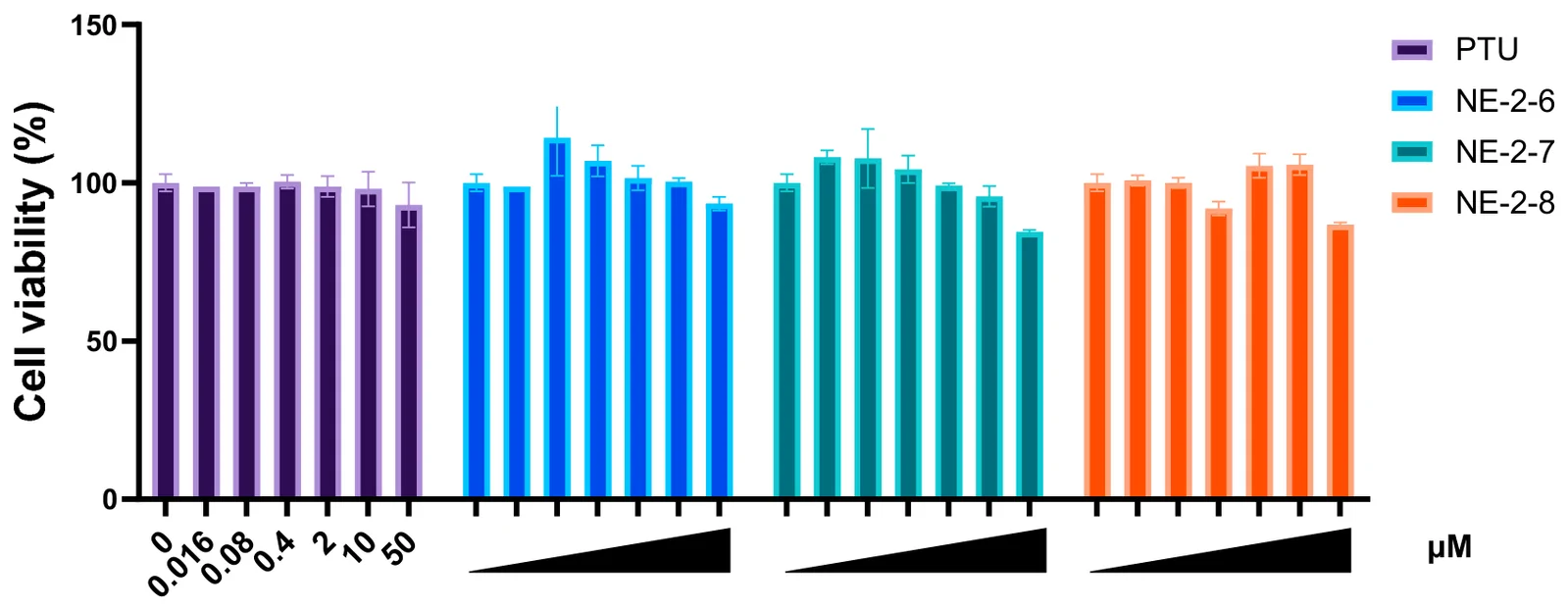
Effects of PTU and NE-2 derivatives on the viability of SNU-790 cells. SNU-790 cells were treated for 24 h with increasing concentrations (0–50 µM) of PTU, NE-2-6, NE-2-7, or NE-2-8, and cell viability was assessed by MTT assay. Cell viability is expressed as a percentage of untreated control cells. Data are presented as mean ± SD of *n* = 3 independent experiments.

**Figure 7:**
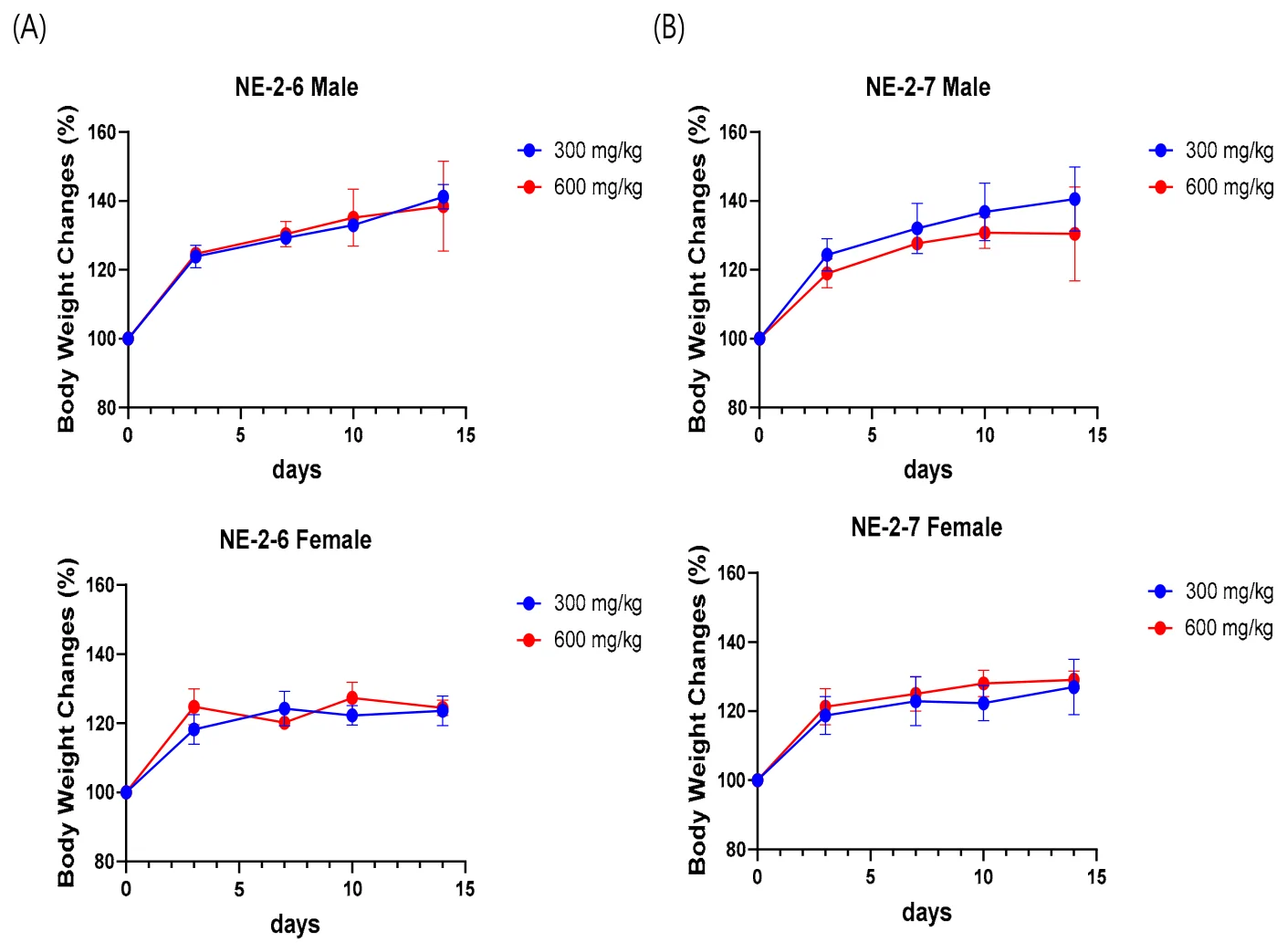
Effects of repeated oral administration of NE-2-6 and NE-2-7 on body weight in mice. Six-week-old male and female ICR mice were treated once daily by oral gavage with NE-2-6 or NE-2-7 (300 or 600 mg/kg) for 14 days, and body weight was recorded at the indicated time points. Body weight is expressed as a percentage of the initial body weight (day 0). Data are presented as mean ±SD (*n* = 3 animals per group). Because a vehicle-only control group was not included, these data should be interpreted as a preliminary comparison between the 300 and 600 mg/kg dose groups rather than as a definitive toxicity assessment. Panels (**A**) and (**B**) represent NE-2-6 and NE-2-7, respectively.

To assess cytotoxicity under the experimental conditions used in this study, MTT assays were performed in SNU-790 cells treated with PTU, NE-2-6, NE-2-7, or NE-2-8 at concentrations ranging from 0.01 to 50 µM for 24 h (Figure 6). The NE-2 derivatives maintained more than 90% cell viability across the tested concentration range, indicating no overt cytotoxicity under these in vitro assay conditions. Short-term tolerability was evaluated following repeated oral administration of NE-2-6 or NE-2-7 at 300 and 600 mg/kg. Because a vehicle-only control group was not included, these data were interpreted as a preliminary dose-comparison observation rather than a definitive toxicity assessment. During the observation period, no mortality, marked body-weight loss, or obvious behavioral abnormalities were observed in either dose group, and body-weight profiles were generally comparable between the 300 and 600 mg/kg groups (Figure 7A,B). These findings indicate no obvious dose-dependent intolerance under the tested short-term conditions; however, the absence of a vehicle control precludes definitive conclusions regarding systemic safety.

**Figure 8:**
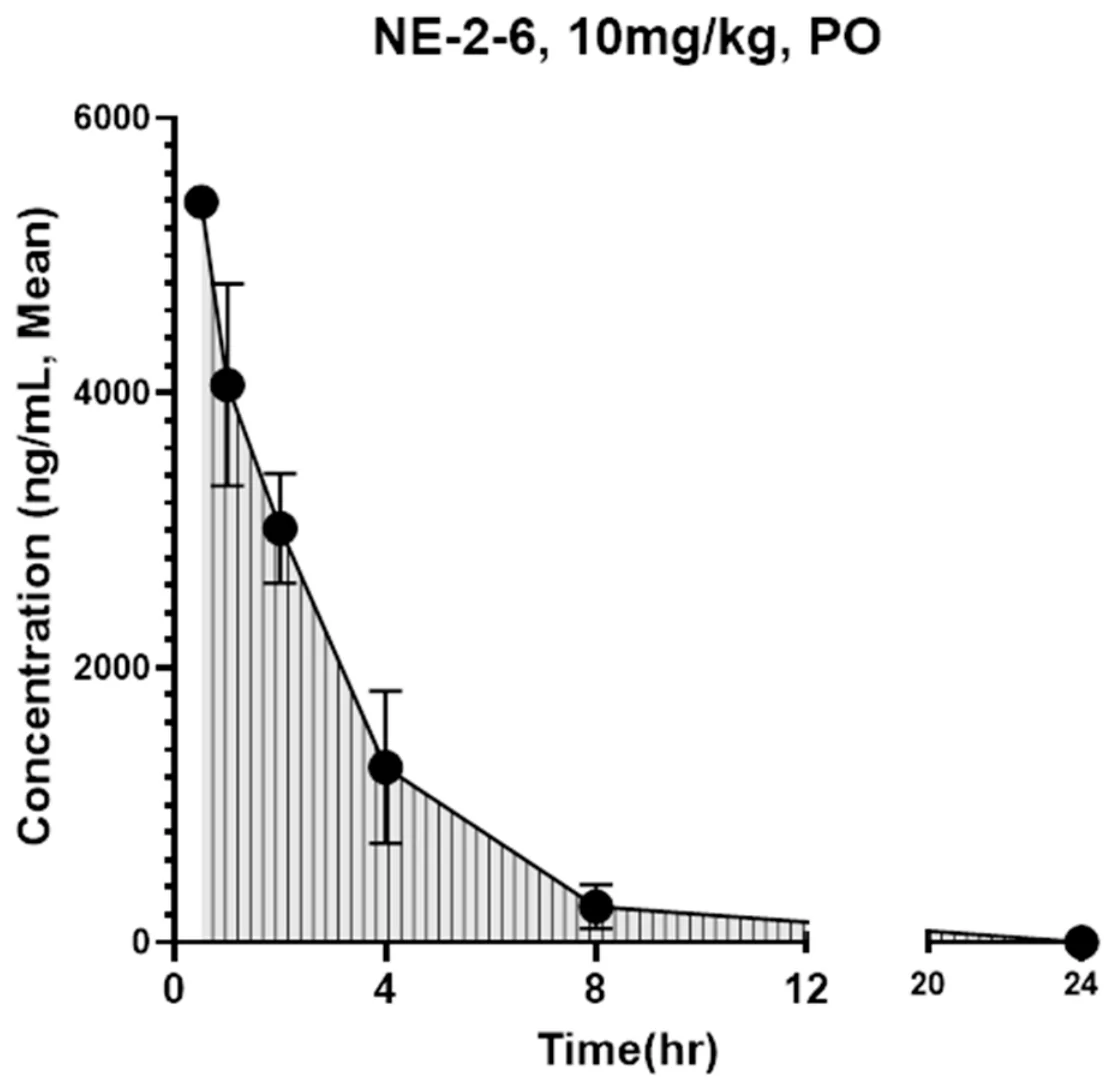
Plasma concentration-time profile of NE-2-6 following single oral administration in mice. NE-2-6 was administered orally to male ICR mice at a single dose of 10 mg/kg, and plasma samples were collected at the indicated time points. Plasma concentrations of NE-2-6 were quantified by UHPLC-MS/MS and are presented as mean ± SD (n = 3 animals per time point). NE-2-6 reached Cmax at approximately 0.5 h, followed by a decline with an apparent terminal half-life of 1.71 h. Plasma exposure demonstrates systemic availability after oral dosing but does not establish thyroid target-site exposure or intrathyroidal TPO engagement.

After a single oral dose of NE-2-6 at 10 mg/kg, plasma concentrations increased rapidly, reaching a Cmax of 5391.27 ng/mL at 0.5 h (Tmax), and then declined with an apparent terminal half-life (t1/2) of 1.71 h (Table 4, Figure 8). The AUClast and AUCinf values were 14,609.55 and 15,253.50 ng·h/mL, respectively, and only 4.22% of the total AUC was extrapolated beyond the last measurable time point. Although plasma concentrations exceeded the in vitro TPO inhibitory IC_50_, plasma exposure alone does not establish thyroid target-site exposure, free intrathyroidal drug concentration, or apical TPO engagement. Therefore, the PK data are interpreted as evidence of systemic oral exposure, whereas the PK–PD relationship requires further thyroid-specific distribution and target-engagement studies.

### 3.6. NE-2-6 Reduces Serum T4 in an Ad-TSHR289-Induced Thyroid Hyperfunction Model

**Figure 9:**
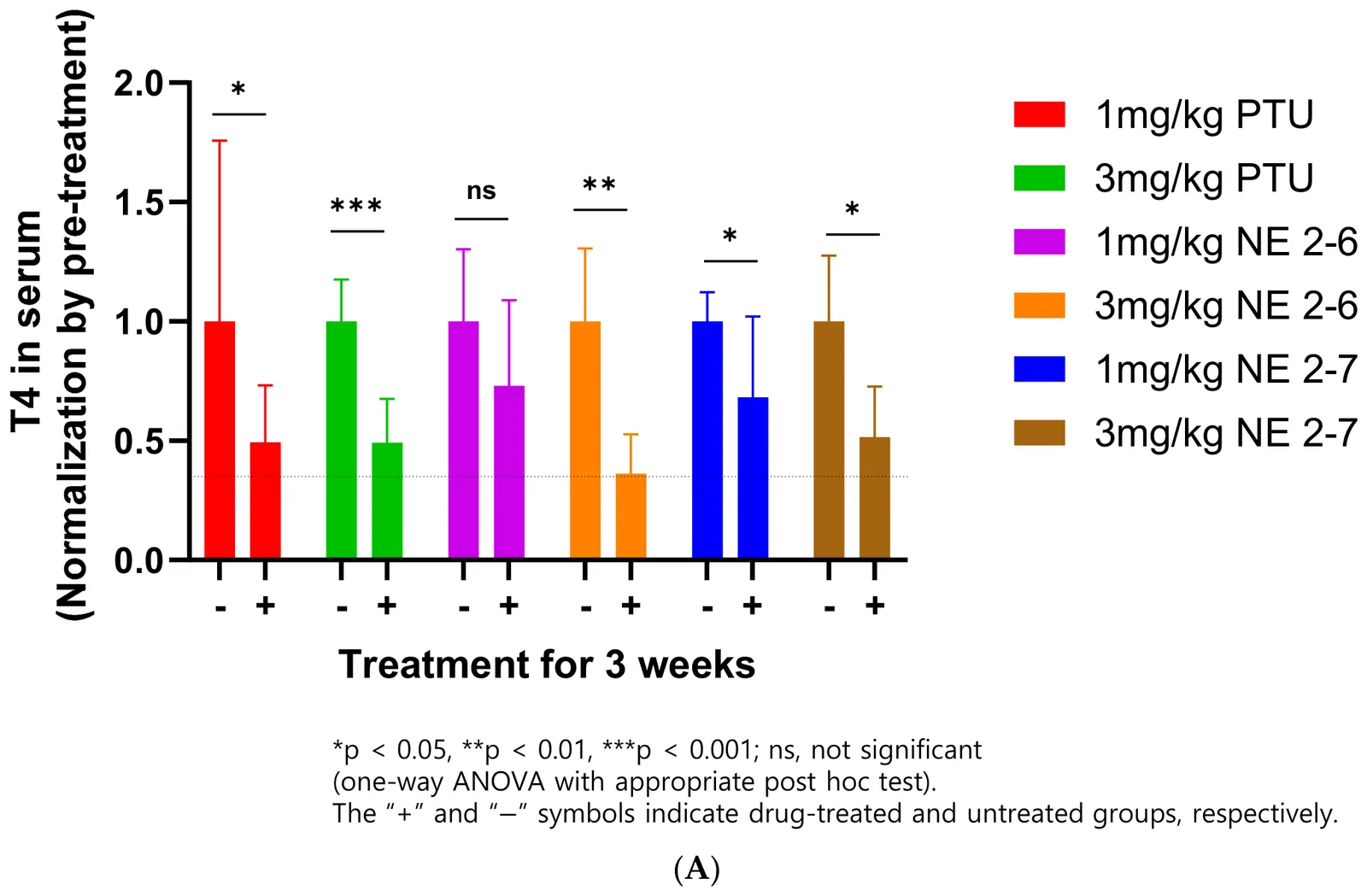

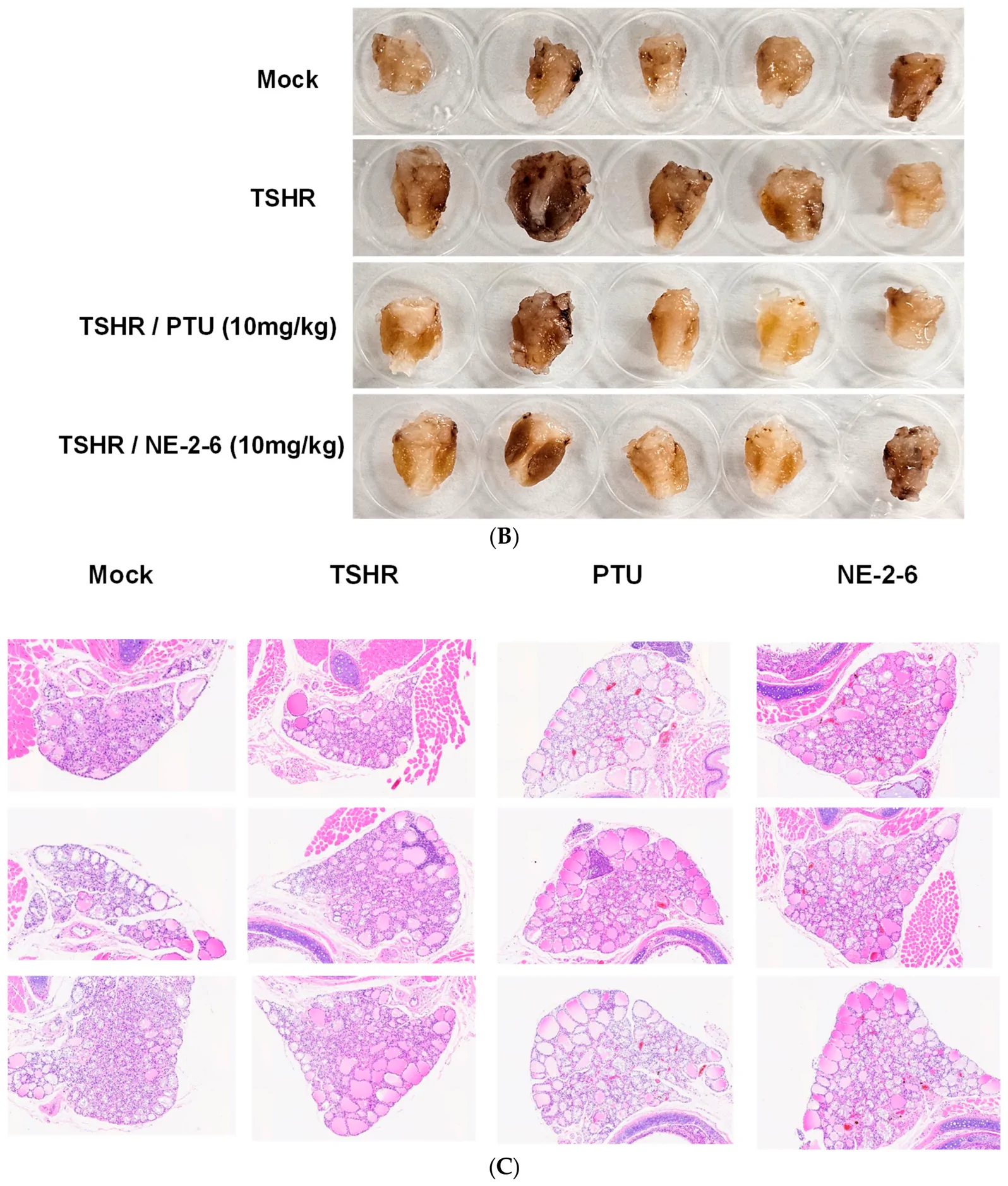
Antithyroid pharmacodynamic effects of NE-2 derivatives in Ad-TSHR289-immunized mice. (**A**) Mice were immunized with Ad-TSHR289 to induce thyroid hyperfunction as described in Materials and Methods and were then treated orally with PTU (1 or 3 mg/kg), NE-2-6 (1 or 3 mg/kg), or NE-2-7 (1 or 3 mg/kg) once daily for 3 weeks. Serum T4 concentrations were measured at the end of the treatment period and normalized to the respective pre-treatment values. Bars represent mean ± SD (*n* = 6 animals per group). Statistical significance was determined by one-way ANOVA followed by Dunnett’s multiple-comparison test versus the Ad-TSHR289 vehicle group. * *p* < 0.05, ** *p* < 0.01, *** *p* < 0.001; ns, not significant. The “+” and “−” symbols indicate the presence and absence of the indicated drug treatment, respectively. (**B**) Mice were immunized with Ad-TSHR289 to induce thyroid hyperfunction and were orally treated once daily with vehicle (TSHR), PTU (10 mg/kg), or NE-2-6 (10 mg/kg) for 3 weeks; untreated, non-immunized mice served as mock controls. Representative thyroid glands from each group (*n* = 6 animals per group) are shown as qualitative morphological observations of thyroid appearance across groups. (**C**) Representative H&E-stained thyroid sections from mock control, Ad-TSHR289 vehicle-treated, PTU-treated, and NE-2-6-treated mice. These representative sections are presented as qualitative supportive observations and do not establish statistically quantified histological improvement or autoimmune disease modification.

To evaluate antithyroid pharmacodynamic activity in vivo, we used an Ad-TSHR289-induced mouse model characterized by elevated serum T4 levels and thyroid hyperfunction-associated changes. Following confirmation of thyroid hyperfunction, animals were treated orally with PTU, NE-2-6, or NE-2-7 for 21 days, after which serum and thyroid tissues were analyzed (Figure 9A). NE-2 derivatives reduced total serum T4 levels relative to the vehicle-treated Ad-TSHR289 group. Among the tested compounds, NE-2-6 produced the most pronounced reduction at 3 mg/kg, lowering circulating T4 concentrations toward the range observed in control animals.

Gross thyroid images and H&E-stained thyroid sections were evaluated as qualitative morphological observations. Representative images suggested thyroid enlargement and discoloration in Ad-TSHR289-immunized mice, whereas thyroid glands from NE-2-6-treated mice appeared less enlarged (Figure 9B). Representative H&E-stained sections from vehicle-treated Ad-TSHR289 mice showed follicular epithelial hyperplasia and reduced colloid, whereas sections from NE-2-6-treated mice appeared to show partial preservation of follicular organization and colloid (Figure 9C). Because thyroid wet weight, standardized colorimetric analysis, and blinded histomorphometric scoring were not performed, these morphological findings are interpreted as qualitative supportive observations rather than statistically quantified endpoints.

Because anti-TSHR antibody titers, TSI activity, thyroid-infiltrating immune-cell subsets, and inflammatory cytokine profiles were not comprehensively assessed, the in vivo data should not be interpreted as evidence that NE-2-6 modifies the autoimmune pathogenesis of Graves’ disease. The observed reduction in serum T4 is consistent with an antithyroid pharmacodynamic effect but does not by itself distinguish direct TPO inhibition from potential effects on TSHR signaling or autoimmune responses.

## 4. Discussion

In this study, we identified and characterized NE-2 derivatives as thyroid peroxidase (TPO) inhibitor candidates through an integrated preclinical evaluation strategy. Among the tested derivatives, NE-2-6 showed the most consistent pharmacological profile, including potent TPO inhibition, relative selectivity over MPO and LPO within the tested peroxidase panel, measurable systemic exposure after oral administration, and antithyroid pharmacodynamic activity in an Ad-TSHR289-induced thyroid hyperfunction model. These findings support NE-2-6 as a lead compound for further evaluation as a TPO-targeting antithyroid candidate.

A major finding of this study is the potent TPO inhibitory activity of NE-2-6. Compared with the parent NE-2 scaffold and the other optimized derivatives, NE-2-6 showed the strongest TPO inhibition while displaying lower activity against MPO and LPO in the tested enzyme panel. This relative selectivity is relevant because TPO is a key enzyme in iodide oxidation and thyroid hormone biosynthesis. Although selectivity over MPO and LPO alone does not establish systemic safety or comprehensive off-target selectivity, it indicates that modification of the NE-2 scaffold improved its preference for TPO inhibition. NE-2-6 therefore represents a useful chemical scaffold for further optimization of TPO-targeting compounds.

The additional biochemical experiments performed in this study provide additional information on the inhibitory behavior of NE-2-6. Molecular docking suggested that NE-2-6 may be accommodated within a catalytic-channel-associated region of TPO [15]. We performed UV–visible spectral scanning, reaction-time-dependent IC_50_ analysis, and H_2_O_2_-dependent inhibition assays to explore the biochemical basis of inhibition. NE-2-6 altered the TPO-associated absorbance profile in the heme/Soret-region range, suggesting that the compound affects the heme-associated catalytic environment or redox state of TPO. In addition, increasing H_2_O_2_ concentration did not reduce the inhibitory effect of NE-2-6, suggesting that inhibition is not readily overcome by excess H_2_O_2_ under the tested conditions and does not by itself establish simple H_2_O_2_-competitive inhibition. The rightward shift in IC_50_ after prolonged reaction further suggests that NE-2-6-mediated inhibition may be linked to sustained TPO/ H_2_O_2_ turnover, possibly through oxidative conversion, depletion, or reduced effective inhibitor availability during the reaction. Together, these findings indicate perturbation of heme-associated TPO catalysis, although the precise kinetic mode remains to be determined.

In cellular experiments, NE-2-6 treatment was associated with reduced TPO mRNA levels in SNU-790 cells. This result is consistent with compound-associated modulation of thyroid-related pathways and supports the biological relevance of evaluating NE-2-6 in thyroid-derived cells. However, TPO gene expression is closely related to thyroid-cell differentiation status, redox balance, cellular stress, and feedback regulation [16]. Therefore, the observed decrease in TPO transcript abundance should not be interpreted as definitive evidence of direct transcriptional repression by NE-2-6. Rather, it should be regarded as a cellular pharmacodynamic response associated with compound treatment. Future studies evaluating TPO protein abundance, cellular TPO enzymatic activity, promoter activity, thyroid differentiation markers, and stress-response pathways will be needed to determine whether this transcriptional change reflects direct regulation or secondary cellular adaptation.

The in vivo findings further support the antithyroid pharmacodynamic activity of NE-2-6. In the Ad-TSHR289-induced thyroid hyperfunction model, NE-2-6 reduced serum T4 levels and was associated with qualitative thyroid histological differences, including follicular epithelial hyperplasia and reduced colloid appearance [17]. These findings are consistent with an antithyroid pharmacodynamic effect but do not by themselves establish direct in vivo TPO target engagement. Importantly, however, the present study was designed primarily to evaluate thyroid hyperfunction and hormone-lowering activity, not autoimmune disease modification. Because anti-TSHR antibody titers, thyroid-infiltrating immune-cell populations, cytokine profiles, germinal-center responses, and relapse after treatment withdrawal were not comprehensively assessed, the current in vivo results should be interpreted as evidence of antithyroid pharmacodynamic activity rather than as proof that NE-2-6 modifies the autoimmune pathogenesis of Graves’ disease [18].

The pharmacokinetic data should also be interpreted cautiously. The single-dose 10 mg/kg PK study demonstrated measurable systemic oral exposure of NE-2-6. However, plasma exposure was not measured at the 1 and 3 mg/kg doses used in the in vivo efficacy experiment, and steady-state exposure, free drug concentration, thyroid tissue distribution, and intrathyroidal target engagement were not determined. Therefore, a quantitative PK–PD relationship between systemic NE-2-6 exposure and serum T4 reduction cannot be established from the current dataset. Future studies should measure plasma and thyroid tissue concentrations at the pharmacologically active doses, determine plasma protein binding and free drug exposure, and evaluate exposure-response relationships together with hepatic and hematological safety markers.

The preliminary safety-related findings also support further evaluation of NE-2-6, but they should be interpreted cautiously [19]. NE-2 derivatives did not show overt cytotoxicity in SNU-790 cells under the tested experimental conditions, and short-term oral administration was not associated with mortality, marked body-weight loss, or obvious behavioral abnormalities. Because the short-term study lacked a vehicle-only control group, these findings represent preliminary dose-comparison observations and do not establish systemic safety or comparative safety advantages over currently used antithyroid drugs. Given that hepatic injury and hematological toxicity are clinically important liabilities of antithyroid drugs, future studies should include long-term repeat-dose toxicity testing, serum hepatic enzyme and bilirubin measurements, liver histopathology, complete blood count analysis, neutrophil assessment, bone marrow evaluation, and broader off-target profiling before the comparative safety profile of NE-2-6 can be defined [20].

In conclusion, NE-2-6 was identified as a potent TPO inhibitor candidate with relative selectivity within the tested peroxidase panel and serum T4-lowering activity in an Ad-TSHR289-induced thyroid hyperfunction model. Although the present study does not establish a definitive binding mechanism, autoimmune disease-modifying activity, a quantitative PK–PD relationship, or a comprehensive safety profile, the combined enzymatic, cellular, pharmacokinetic, and in vivo findings support further preclinical evaluation of NE-2-6 as a TPO-targeting antithyroid candidate. As a next step, steady-state kinetic analysis using varied iodide and tyrosyl donor concentrations will be important to better understand the mechanism of TPO inhibition by NE-2-6. Direct binding studies, such as SPR or ITC, as well as evaluation of autoimmune endpoints and long-term safety, will also help further characterize the compound.

## Figures and Tables

**Table 1:** The primers used in this study.

Genes	F Primer Sequence (5′ → 3′)	R Primer Sequence (5′ → 3′)
TPO	CACCAGGCTTTCAGCCCAT	GGACAGCACAAAGAGCCTTTCC
TSHR	GAGTTTCCTTCACCTCACACGG	CTGCTCTCATTAACACATCAAGGAC
TG	CCAGTGGCTTCTCTTCCTGACT	CCTTGGAGGAAGCGGATGGTTT
GAPDH(Control)	GTCTCCTCTGACTTCAACAGCG	ACCACCCTGTTGCTGTAGCCAA

**Table 2:** Enzyme activity in the second-round screening of the chemical library.

Chemical (First-Round Lead Candidate)	IC_50_ (µM)	HL-60 MPO Inhibition (%) (Treatment of Cell Lysate)	HL-60 MPO Inhibition (%) (Treatment of Culture Medium)	MTT Assay (Relative Cell Death at 50 µM in HEK293T Cells)	Third-Round Lead Candidate
AZD3241 (PC)	0.021	98	0	6	-
NE-1	0.6	70	20	45	X
NE-2	1.53	95	0	0	O
NE-3	1.98	98	5	0	X
NE-4	1.53	98	0	5	O
NE-5	2	71	15	5	X
NE-6	1.6	98	0	5	O
NE-7	1.24	95	0	5	O
NE-8	0.32	94	0	5	O
NE-9	1.9	90	0	7	X
NE-10	1.97	90	0	3	X
NE-11	1.84	90	0	3	X
NE-12	0.93	90	0	15	X
NE-13	1.02	95	0	5	O
NE-14	1.59	98	0	5	O
NE-15	1.91	80	0	7	X
NE-16	1.64	80	25	35	X

**Table 3:** Peroxidase inhibitory activity and selectivity of NE-2 derivatives.

	Propylthiouracil (PTU)	NE-2-6	NE-2-7	NE-2-8
Molecular weight	170.23	344.38	373.42	359.39
Structure	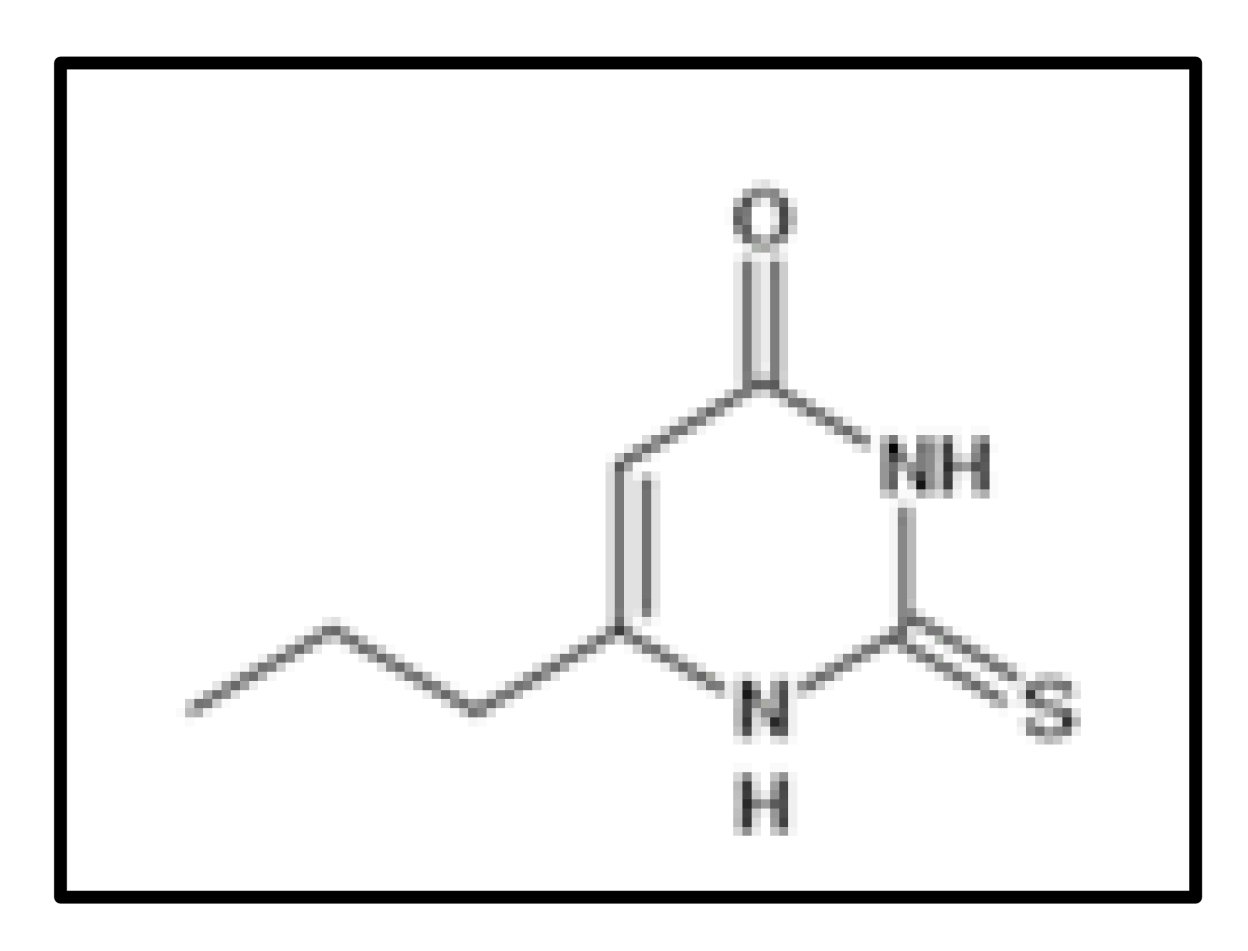	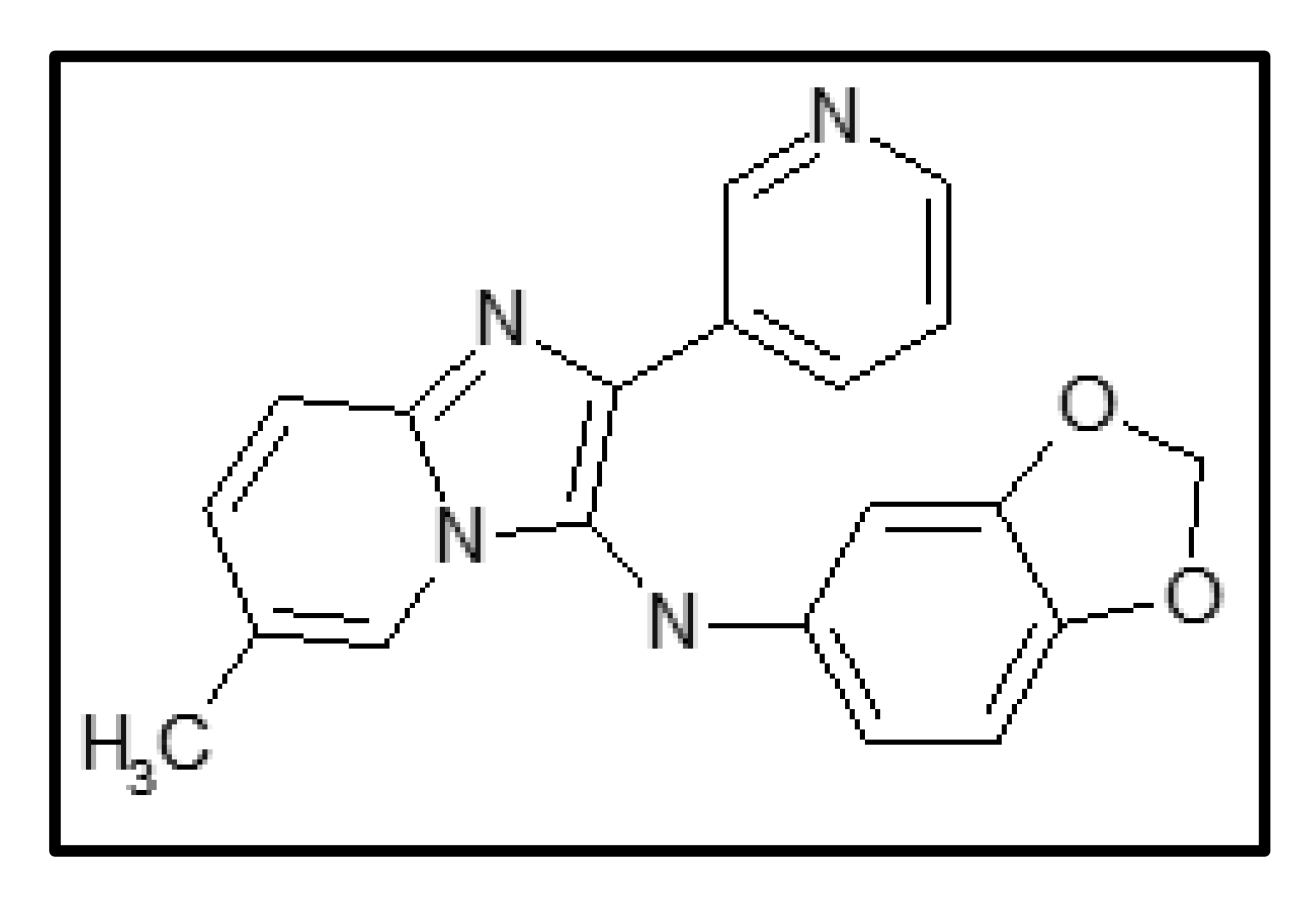	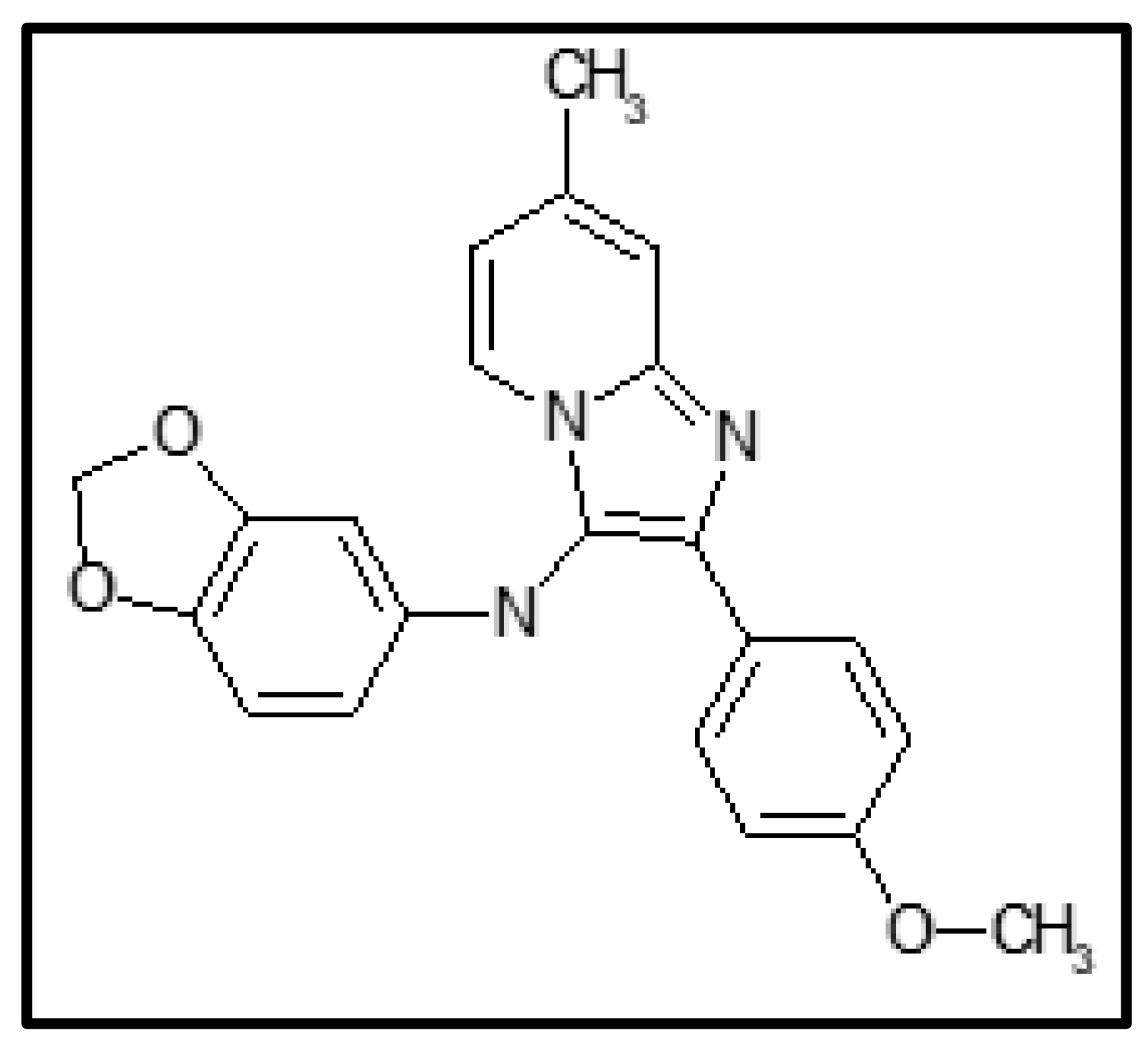	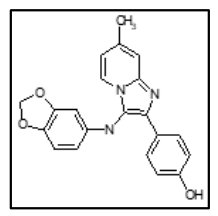
SMILES	N1C(=O)C=C(NC1=S)CCC	N12C=C(C=CC1= NC(C1=CC=CN=C1)=C2NC1=CC= C2OCOC2=C1)C	N12C=C(C=CC1=NC(C1C=CC(=CC=1)OC)=C2NC1=CC=C2C(OCO2)=C1)C	N12C=C(C=CC1=NC (C1C=CC(=CC=1)O)=C2NC1=CC=C2OCOC2=C1)C
MPO IC_50_ (μM)	5.5	6.45	1.5	0.77
LPO IC_50_ (μM)	>50	0.89	2.25	1.05
TPO IC_50_ (μM)	0.119	0.013	0.02	0.016
MPO/TPO (selectivity ratio)	46.2	496.2	75	49.1
LPO/TPO (selectivity ratio)	>420	68.5	112.5	65.6

**Table 4:** Pharmacokinetic parameters of NE-2-6 after single oral administration in mice.

Time (h)	Mean	SD	CV (%)
0.5	5391.270	64.749	1.2
1	4057.645	735.539	18.1
2	3014.117	398.781	13.2
4	1275.579	554.963	43.5
8	261.385	160.390	61.4
24	0.000	0.000	NA
Cmax (ng/mL)	5391.27		
Tmax (h)	0.50		
AUClast (ng·h/mL)	14,609.55		
AUCinf (ng·h/mL)	15,253.50		
t1/2 (h)	1.71		
CL/F (mL/h/kg)	655.59		
Vd/F (mL/kg)	1615.11		
Rsq_adjusted	1.00		
%AUCexp (%)	4.22		

## Data Availability

The original contributions presented in this study are included in the article. Further inquiries can be directed to the corresponding author.
